# An Efficient Approach for Differentiating Alzheimer's Disease from Normal Elderly Based on Multicenter MRI Using Gray-Level Invariant Features

**DOI:** 10.1371/journal.pone.0105563

**Published:** 2014-08-20

**Authors:** Muwei Li, Kenichi Oishi, Xiaohai He, Yuanyuan Qin, Fei Gao, Susumu Mori, for the Alzheimer's Disease Neuroimaging Initiative

**Affiliations:** 1 College of Electronics and Information Engineering, Sichuan University, Chengdu, China; 2 The Russell H. Morgan Department of Radiology and Radiological Science, The Johns Hopkins University School of Medicine, Baltimore, Maryland, United States of America; 3 Department of Radiology, Tongji Hospital, Tongji Medical College, Huazhong University of Science and Technology, Wuhan, China; 4 Shandong Medical Imaging Research Institute, Shandong University, Jinan, China; Wake Forest School of Medicine, United States of America

## Abstract

Machine learning techniques, along with imaging markers extracted from structural magnetic resonance images, have been shown to increase the accuracy to differentiate patients with Alzheimer's disease (AD) from normal elderly controls. Several forms of anatomical features, such as cortical volume, shape, and thickness, have demonstrated discriminative capability. These approaches rely on accurate non-linear image transformation, which could invite several nuisance factors, such as dependency on transformation parameters and the degree of anatomical abnormality, and an unpredictable influence of residual registration errors. In this study, we tested a simple method to extract disease-related anatomical features, which is suitable for initial stratification of the heterogeneous patient populations often encountered in clinical data. The method employed gray-level invariant features, which were extracted from linearly transformed images, to characterize AD-specific anatomical features. The intensity information from a disease-specific spatial masking, which was linearly registered to each patient, was used to capture the anatomical features. We implemented a two-step feature selection for anatomic recognition. First, a statistic-based feature selection was implemented to extract AD-related anatomical features while excluding non-significant features. Then, seven knowledge-based ROIs were used to capture the local discriminative powers of selected voxels within areas that were sensitive to AD or mild cognitive impairment (MCI). The discriminative capability of the proposed feature was measured by its performance in differentiating AD or MCI from normal elderly controls (NC) using a support vector machine. The statistic-based feature selection, together with the knowledge-based masks, provided a promising solution for capturing anatomical features of the brain efficiently. For the analysis of clinical populations, which are inherently heterogeneous, this approach could stratify the large amount of data rapidly and could be combined with more detailed subsequent analyses based on non-linear transformation.

## Introduction

Alzheimer's disease (AD) is the most common neurodegenerative dementia, which causes the gradual loss of cognitive functions. A definite diagnosis of AD can only be made through autopsy findings, such as amyloid deposition and neurofibrillary tangles [1], [2]. In practice, the diagnosis of AD is based on clinical criteria [3]. In addition, findings from neuroimaging technologies, such as magnetic resonance imaging (MRI) [4], positron emission tomography (PET) [5], [6], or single-photon emission computed tomography [7] could further increase the diagnostic accuracy of AD [8]. Among these modalities, structural MRI has been recognized as a marker for neuronal injury, which could be detected as volume loss [9], cortical thinning [10], or changes in shape [11] seen in a set of anatomical structures such as the medial temporal area, the posterior cingulate area, the thalamus, and other cortical areas.

One promising extension of these findings in anatomical MRI is the use in the analysis of large clinical data, in which a large amount of anatomical MRIs of an elderly population, collected through multiple institutes, could be used to evaluate the possibility of AD or to evaluate the future risk for developing dementia, on an individual basis [12]. A range of studies have demonstrated that morphometric features extracted from structural MRI, along with machine-learning techniques, could be used to classify a single subject as a member of a particular clinical category [13]–[17]. One group of these studies considers voxel-based tissue probability maps directly as features in the classification [17]–[20]. Another group focuses on regional characteristics, such as volume, shape, thickness within one single anatomical structure, or the multivariate description over the whole-brain parcels obtained using automated segmentation tools [21]–[25]. The third group first characterizes the shape of an ROI as a series of parameters, such as spherical harmonics or log-Jacobian determinants from tensor-based morphometry, and then utilizes the parameters as features [26], [27]. Some other studies have focused on the combination of multiple modalities, including MRI, PET, and cerebrospinal fluid (CSF), and have yielded good classification accuracies [28]–[30].

The studies mentioned above are often coupled with highly accurate non-rigid registration that, although proven to be effective, is also known to invite several nuisance factors, such as transformation parameter dependency, computational complexity, and uncertainty in the range of applicability with respect to the degree of anatomical abnormality; the transformation accuracy may vary depending on the anatomical difference between the atlas and patients. For example, in a routine voxel-based pipeline, there are parameters that control the elasticity and smoothness of the deformation field used for transforming target images to a standard space. The choice of these parameters is usually pre-fixed regardless of anatomical differences between the groups. The statistical analysis based on the large number of voxels (typically more than one million) also poses a serious challenge for subsequent correlation analyses with diagnosis and other types of clinical information. A scheme to contract anatomical features to a much more manageable size seems essential [12], [31].

In a previous study, a residual-based measurement using an atlas grid was reported, which could successfully capture anatomical features of various types of neurodegenerative diseases [32]. This approach was named the Gross feature recognition of Anatomical Images based on Atlas grid (GAIA), which is a highly time-efficient method for the image recognition and does not rely on non-linear transformation. In this approach, an atlas with more than 200 pre-defined structures was linearly superimposed on a target image and the intensities of the defined structures were measured. The intensity rankings of the defined structures were then used as anatomical features. Anatomical alterations beyond the normal range would lead to gross misregistraion and abnormal intensities of the defined structures, which was captured as an anatomical feature. Although utilization of the pre-defined atlas grid (i.e., anatomical structure parcellation map) is an effective way for dimensional reduction [33], one of the limitations of GAIA is reduced sensitivity to localized anatomical alterations that only affect part of a pre-defined structure [34].

In our study, we extended the GAIA approach to voxel-based feature recognition, in which, instead of applying a pre-defined atlas grid for feature extraction and reduction, we employed data-specific and knowledge-based masks. These masks were created based on voxel-based statistics results and the Disease-Specific Anatomical Filtering method [35]. Because GAIA relies on image intensities, standardization of voxel intensity values across different images is one of the technical challenges. To standardize the intensity of MRI images, histogram equalization, in which the tonal distribution of an input image and a template are mathematically matched, is often used [36]. However, the spatial relationship between pixels in the target image and the template is disregarded in this approach, which sometimes leads to artifacts caused by the increased contrast-to-noise ratio in low-intensity areas. Therefore, for voxel-based analysis, we introduced the local binary pattern (LBP), which has been widely used in various applications and has been proven robust to monotonic gray-level changes, and is also computationally efficient [37], [38]. A frequent application of LBP is facial recognition attributed to its invariance to illumination changes in facial pictures. Similarly, cross-scanner variability in MRI images can also be characterized as a monotonic change, where the ranking value of the average intensity in a particular anatomical tissue would not change over subjects. For instance, in T1-weighted images, the intensities of gray matter pixels are always lower than those of white matter pixels in an image retrieved from any scanner.

A total of 363 multicenter subjects from the ADNI database were recruited in the present study in order to validate the feasibility of using gray-level invariant features for classification. A machine-learning tool, namely, a support vector machine (SVM) [13]–[17] was utilized to investigate the discriminative capability of the proposed features extracted from those subjects. Specifically, SVM was trained on a subgroup of subjects with clinically-labeled features, and then was tested by cross-validation using another subgroup of subjects with features blinded from their labels [39]. Feature selection based on statistical methods was implemented to LBP-TOP (three orthogonal planes) maps to exclude disease-unrelated features and accelerate the training procedure. In addition, seven pre-defined custom masks were designed to investigate the discriminative powers of local features within areas that are sensitive to AD, including the hippocampus, amygdala, the parahippocampal gyrus, the entorhinal area, the temporal lobe, the lateral ventricle, and an overall mask that combined the six masks. The selected features, along with the knowledge-based masks, present a possible approach to build disease-specific filters, particularly on a linear transformation basis.

## Method

### Data description

Data used in the preparation of this article were obtained from the Alzheimer's Disease Neuroimaging Initiative (ADNI) database (adni.loni.usc.edu). The data were analyzed anonymously, using publicly available secondary data from the ADNI study; therefore, no ethics statement is required for this work. The ADNI was launched in 2003 by the National Institute on Aging (NIA), the National Institute of Biomedical Imaging and Bioengineering (NIBIB), the Food and Drug Administration (FDA), private pharmaceutical companies, and non-profit organizations, as a $60 million, five-year public-private partnership. The primary goal of ADNI has been to test whether serial magnetic resonance imaging (MRI), positron emission tomography (PET), other biological markers, and clinical and neuropsychological assessment can be combined to measure the progression of mild cognitive impairment (MCI) and early Alzheimer's disease (AD). The determination of sensitive and specific markers of very early AD progression is intended to aid researchers and clinicians in developing new treatments and monitoring their effectiveness, as well as lessening the time and cost of clinical trials.

The Principal Investigator of this initiative is Michael W. Weiner, MD, VA Medical Center and University of California – San Francisco. ADNI is the result of the efforts of many co-investigators from a broad range of academic institutions and private corporations, and subjects have been recruited from over 50 sites across the U.S. and Canada. The initial goal of ADNI was to recruit 800 subjects, but ADNI has been followed by ADNI-GO and ADNI-2. To date, these three protocols have recruited over 1500 adults, ages 55 to 90, to participate in the research, consisting of cognitively normal older individuals, people with early or late MCI, and people with early AD. The follow-up duration of each group is specified in the protocols for ADNI-1, ADNI-2, and ADNI-GO. Subjects originally recruited for ADNI-1 and ADNI-GO had the option to be followed in ADNI-2. For up-to-date information, see www.adni-info.org.

The key eligibility criteria used in ADNI was detailed at http://www.adniinfo.org/Scientists/ADNIGrant/ProtocolSummary.aspx. Briefly, subjects with mini-mental state examination (MMSE) [40] scores between 20–26 (inclusive), a clinical dementia rating (CDR) [41] of 0.5 or 1.0, and who met the NINCDS/ADRDA criteria [42] for probable AD and were diagnosed as AD. The diagnosis of MCI was made if the subjects had MMSE scores between 24–30 (inclusive), a memory complaint, had objective memory loss measured by education-adjusted scores on the Wechsler Memory Scale Logical Memory II [43], a CDR of 0.5, the absence of significant levels of impairment in other cognitive domains, essentially preserved activities of daily living, and an absence of dementia. Normal controls followed the criteria: MMSE scores between 24 and 30 (inclusive), a CDR of 0, non-depressed, non-MCI, and non-demented. The age range of normal subjects is roughly matched to that of MCI and AD subjects. Therefore, there should be a minimal enrollment of normal subjects under the age of 70.

A total of 363 subjects from the ADNI1 (1.5T) database, with corresponding baseline MRIs, were used in this study. Structural MRIs were acquired from 1.5 T scanners across 51 centers with a protocol individualized for each scanner, as defined in http://adni.loni.usc.edu/. Images were downloaded from https://ida.loni.usc.edu/ in NiFTI formats with geometry distortion corrected and B1 correction. The individuals analyzed in this study included: 80 patients with probable AD (38 males, 42 females, age±SD = 77.1±5.5 years; MMSE±SD = 23.1±1.9), 141 patients with MCI (80 males, 61 females, age±SD = 75.7±6.4 years; MMSE±SD = 27.0±1.6), and 142 normal elderly controls (73 males, 69 females, age±SD = 76.5±7.2 years; MMSE±SD = 29.2±0.93). All subjects studied in this work were followed up for three years and all MCI patients were clinically stable during their last visits (month 36). The demographics and characteristics of the selected population are shown in Table 1, together with their between-group differences in age, MMSE and sex.

**Table 1:** The demographics and characteristics of the selected population.

Group	Number	Sex	Age	MMSE	Number of scanning protocols
AD	80	38M/42F	77.1±5.5 [57–89]	23.1±1.9 [20]–[26]	42
MCI	141	80M/61F	75.5±6.4 [57–90]	27.0±1.6 [24]–[30]	47
NC	142	73M/69F	76.5±7.2 [57–92]	29.2±0.9 [25]–[30]	50
					
Differences					
AD vs. NC	/	P = 0.675	p>0.05	p<0.0001	
MCI vs. NC	/	P = 0.404	p>0.05	p<0.0001	

The between-group differences in age and MMSE were assessed with the student's t-test. The differences in gender were evaluated by a two-sided Pearson Chi-Square test.

### Preprocessing

The structural MRI images were first skull-stripped using a Matlab suite called SPM8 [44]. To be specific, a brain mask was obtained for each subject by combining three individual tissue probability maps, including white matter, gray matter, and cerebrospinal fluid (CSF), obtained from the unified segmentation module incorporated in SPM. The mask was then superimposed on the original image to clean up tissues outside the brain, such as the skull, skin, and neck. Skull-stripped images were then co-registered (linear transformed) to a template, namely EVE [45], using 12 degrees of freedom (DOF) affine [46] to standardize each individual to the Montreal Neurological Institute (MNI) space [47]. To obtain an unbiased co-registration, 12 degrees of freedom affine were employed with cost function setting to mutual information (MI), which was proved robust to inter-subject intensity variations [48]. After the co-registration, spatial locations and global brain sizes, which were considered as covariates in analyzing the disease-specific features, were normalized for all these subjects.

### Gray-level invariant features

**Figure 1:**
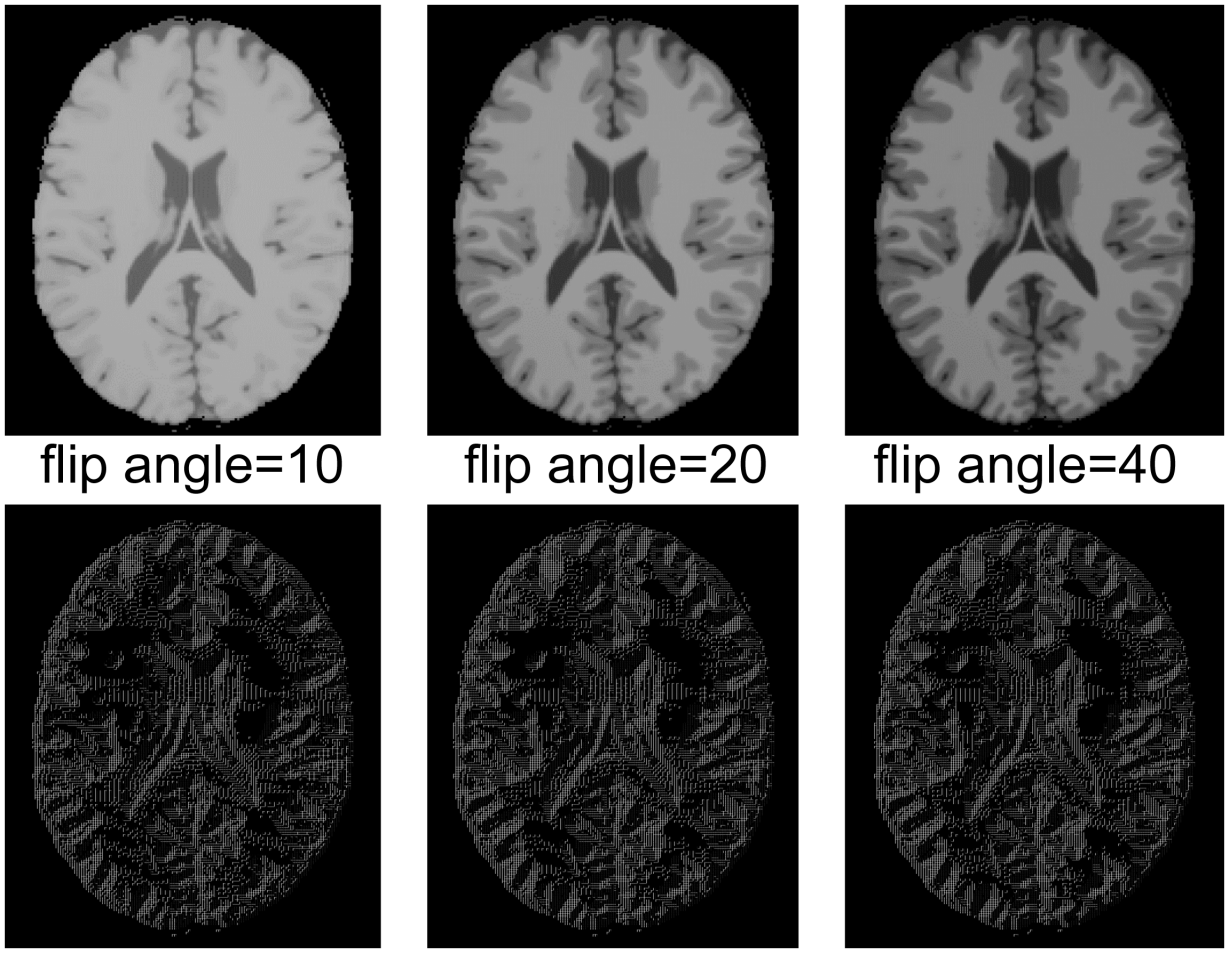
A 2D LBP test on simulated MRIs. The first row displays the MRI images and the second row displays their corresponding LBP maps. Images scanned with different flip angles are shown in columns.



**Figure 2:**
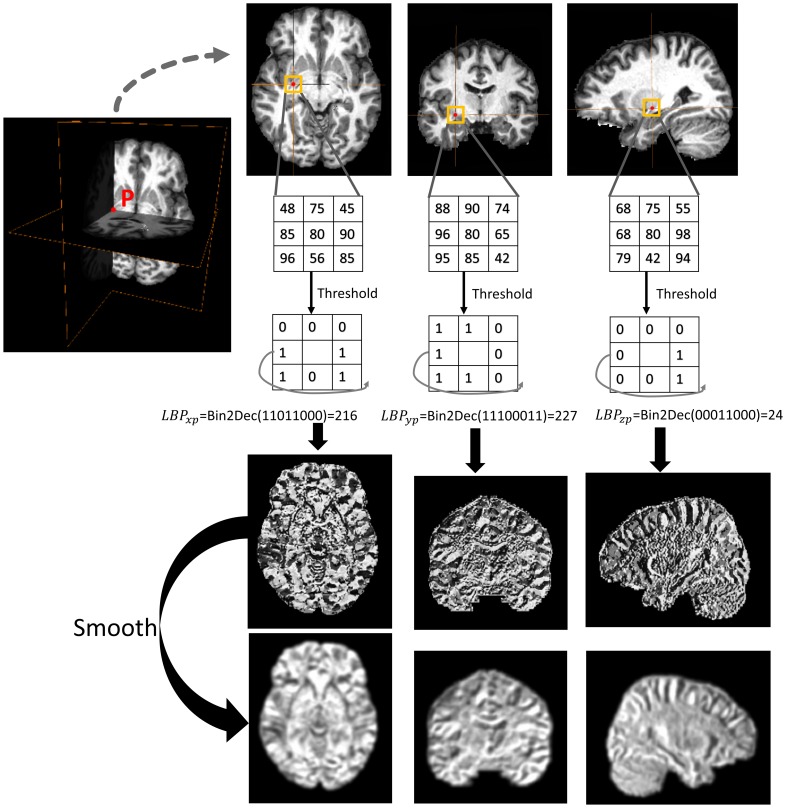
A brief illustration of the calculation of LBP-TOP value in pixel p in the axial, coronal, and sagittal orientations. Pixel p is denoted by the red color, with its 3×3 neighborhood circled by a yellow square in the 2D plane. Bin2Dec is a function for transferring binary code to decimal values.

LBP operator was used to represent the gray-level invariant features of the original image with low computational complexity. It described the local structure by thresholding the intensities of a set of P neighboring pixels *set(I_P_)* with the intensity of its center pixel *I_C_*, and then represented the feature as a binary code, as explained in (1). A demonstration of its gray-level invariance is shown in Fig 1, where LBP is applied to 2D phantom MRIs with multiple monotonic gray-level changes. MRI images shown in the first row of Fig 1 were simulated by BrainWeb [49] by setting the simulated Flip Angle to 10, 20, and 40 respectively. The second row shows corresponding LBP maps, which, as expected, differed little from each other. The reason for using phantom images is to guarantee that all images were exactly in the same coordinate.

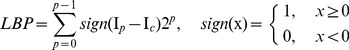
(1)Rotation invariant LBP was an extended version of the original operator with robustness to image rotation [50]. Given that affine has been applied to exclude the rotation influence, traditional LBP operator was deemed competent for feature extraction in the present study, which resulted in 256 possible labels within a 3×3 neighborhood. In this case, the intensity of the LBP map ranged from 0–255 in a 2D image. A straightforward 3D LBP form, namely LBP-TOP (three orthogonal planes), was proposed in a previous study to describe spatiotemporal signals of facial expression by simply concatenating features extracted from three orthogonal 2D planes [51]. In the present study, LBP-TOP operator traversed all 3×3 neighborhoods in every 2D slice varying separately along axial, coronal, and sagittal orientations, as shown in Fig 2. Thus, every pixel p was potentially represented by a 3D vector [*LBP_xp_ LBP_yp_ LBP_zp_*], denoting the LBP value separately on the y-z, x-z, and x-y planes.

Furthermore, three types of features were evaluated in this study for comparison, including Type 1 images without any intensity adjustment, Type 2 histogram-equalized images, and the proposed Type 3 LBP-TOP maps. Note that all features were based on images transformed to a standard space through affine co-registration.

### Two-step feature selection

Feature selection is known as a process of selecting an important subset of features for model construction in machine-learning. In our study, a feature vector, which was used to represent the anatomical attribute of a brain, recorded the voxel intensities of its LBP maps. However, not all voxels encoded useful information for the recognition of certain anatomical features. The most relevant voxels could be retained according to several criteria; for example, voxels behaved significantly statistically differently between the normal group and the disease group, or voxels located in certain areas associated with previous pathological evidence.

**Figure 3:**
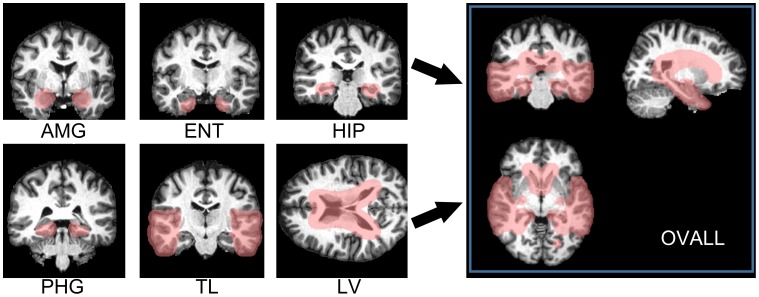
Seven knowledge-based masks customized for capturing AD-specific morphometry. These masks covered areas that have been consistently demonstrated as sensitive to AD, including the amygdala (AMG), the entorhinal area (ENT), the hippocampus (HIP), the parahippocampal gyrus (PHG), the temporal lobe (TL), the Lateral ventricle (LV), and a mask that combined the aforementioned six masks (OVALL).

The LBP map represented an approximation of the shape of high-gradient areas, such as boundaries or corners, but also noise in MRIs. When implementing feature selection directly on LBP maps, limited numbers of usable features are sometimes suppressed by imaging noise. Thus, as shown in Fig 2, all LBP maps were smoothed using a Gaussian filter specifying the full width half maximum (FWHM) to 4 mm×4 mm×4 mm. This preprocessing was then followed by a two-step feature selection known as data-driven selection and knowledge-driven selection. The data-driven selection applied a two-sample t-test on a voxel basis over the entire brain to retain features that showed statistical differences between the AD/MCI and NC groups. It has been suggested in the existing literature [35], [52], [53] that correctly reducing the number of features, can accelerate computation and improve performance by selecting features with the greatest discriminative power. In this study, features with significant differences (p<0.001, uncorrected) between the patient group and the normal group were selected, only within the training samples. The second step is known as a knowledge-driven selection, where priori masks were customized to select disease-specific areas that were expected to have the greatest positive contributions to the classification. Note that the second step was applied to voxels selected by the first-step selection. As shown in Fig 3, seven binary masks (selected area = 1, background = 0) that stood in the same coordinates with EVE were built according to anatomical knowledge for encoding morphometric changes over groups. It was expected that the boundaries of the masks eased enough to cover variant types of non-rigid morphometry over subjects within their corresponding anatomical structures, including the amygdala (AMG), the entorhinal area (ENT), the hippocampus (HIP), the parahippocampal gyrus (PHG), the temporal lobe (TL), the lateral ventricles (LV), and an overall mask (OVALL) built by executing an “OR” operation on the six masks mentioned above. These were the areas that had consistently certified values for differentiating AD/MCI from normal states. Specifically for LBP maps, two-step feature selection was carried out separately on three orthogonal planes, and then, the three individual parts were concatenated into a single feature vector (Type 3 feature). Therefore, one selected voxel at coordinate (x, y, z) satisfied two requirements: 1) voxels showed significant inter-group differences in intensities at (x, y, z) over all studied images; 2) intensity = 1 on the binary mask at (x, y, z). Note that independent selections were carried out with different masks in the second step. In other words, each mask yielded a particular feature vector to describe the entire brain. Potentially, AD-specific filters can be derived from the two-step feature selection specific to linearly transformed voxels. For comparison, Type 1 and Type 2 features were also extracted from intensity-unadjusted images and histogram-equalized images, which were then subsequently refined through similar strategies used for feature selection.

### Validation of the proposed feature

The discriminative value of three types of features were studied and compared in terms of the performances of classifiers employing these features separately. The feature matrix with selected features of each training sample listed in rows was sent to the SVM program [54] with their clinical labels (AD  =  positive  = 1, NC  =  negative  = −1). SVM, first developed by Vapnik in 1995 [55], was designed for the classification of non-linear and high-dimensional data. Therefore, it was compatible with image -based recognition in various biomedical applications. In the general SVM process, a classifier was trained by mapping the input *m*-dimensional feature vectors into *l*-dimensional space (*l>m*) using kernel functions. SVM aimed to find the maximum-margin hyperplane that represented the largest separation or margin between the two clinical groups in the feature space. The boundaries of the hyperplane were represented by the support vectors, equivalent to the training samples on the margins. After this process, the trained classifier could be used to map incoming testing data into the *l*-dimensional feature space and thereafter assigned it to the appropriate category. An unbiased estimate of true classification performance was obtained by employing 10-fold cross-validation that initially divided all samples to 10 subsets and then iteratively left one subset out of training for subsequent testing until each of the 10 subsets were validated. To avoid possible bias, each cross-validation process was repeated 30 times, and a mean estimation of classification performance was obtained. Note that t-test-based feature selection (first-step selection) was also constrained in the cross-validation loop; that is, the testing sample was not part of the two-sample t-test to avoid over-fitting of the classifier. The classification accuracies of classification models using different types of features were analyzed and compared in terms of classification accuracy (percentage of correctly classified subjects), specificity, sensitivity, receiver operating characteristic (ROC) curve [56], [57], as well as area under ROC curve (AUC), where AUC  = 0.5 stands for completely random predictions and AUC  = 1.0 stands for perfect separation [58]. The computation time of the proposed model was also studied and compared to the pipelines using volume, shape, and thickness –based features.

## Results

### Biological sensitivity of the gray-level invariant feature

**Figure 4:**
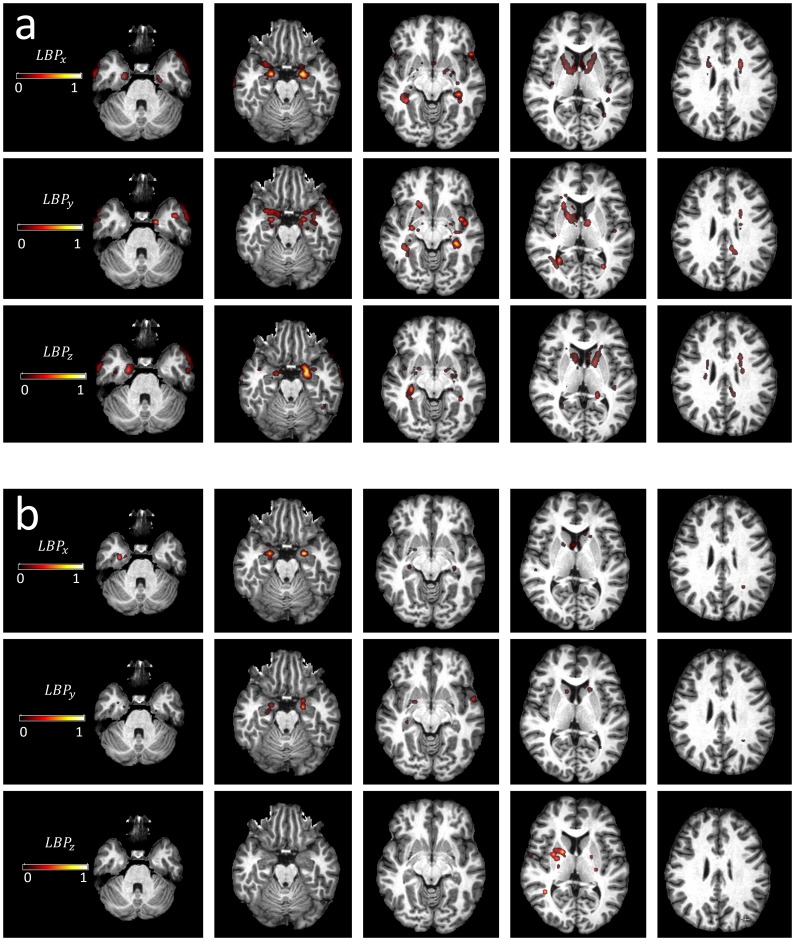
Average feature weights yielded by linear SVM in differentiating a) AD from NC, and b) MCI from NC. In either a) or b), average weights separately obtained from three orthogonal planes are displayed in rows, and different slices along the axial orientation are displayed in columns. The ascending weights are shown from darkness to brightness.

To investigate whether our classification model based on the LBP and SVM could give appropriate weighting to the known anatomical structures involved in AD to separate AD or MCI from NC, the weights of training features derived from the linear SVM output were mapped onto the template space. Specifically, in every cross-validation procedure with one subset of samples left out for training, each variable of the feature vector was assigned a weight calculated by training on the remnant nine subsets of the samples. The average weight for the same variable was then calculated by averaging the weights produced from 10 iterations of cross-validations. Fig 4 shows feature weights, which have been normalized to [0, 1], obtained from three LBP-TOP maps separately. Synthetically, AD and MCI show similar patterns of discriminative power over the brain where highly-weighted areas by SVM are: the hippocampus; the amygdala; the putamen; the thalamus; the insula; the precuneus; the anterior cingulate gyrus; the posterior cingulate gyrus; the areas around the lateral ventricles; and several areas in the temporal lobe.

### Discriminative powers of selected features within seven knowledge-based masks

**Figure 5:**
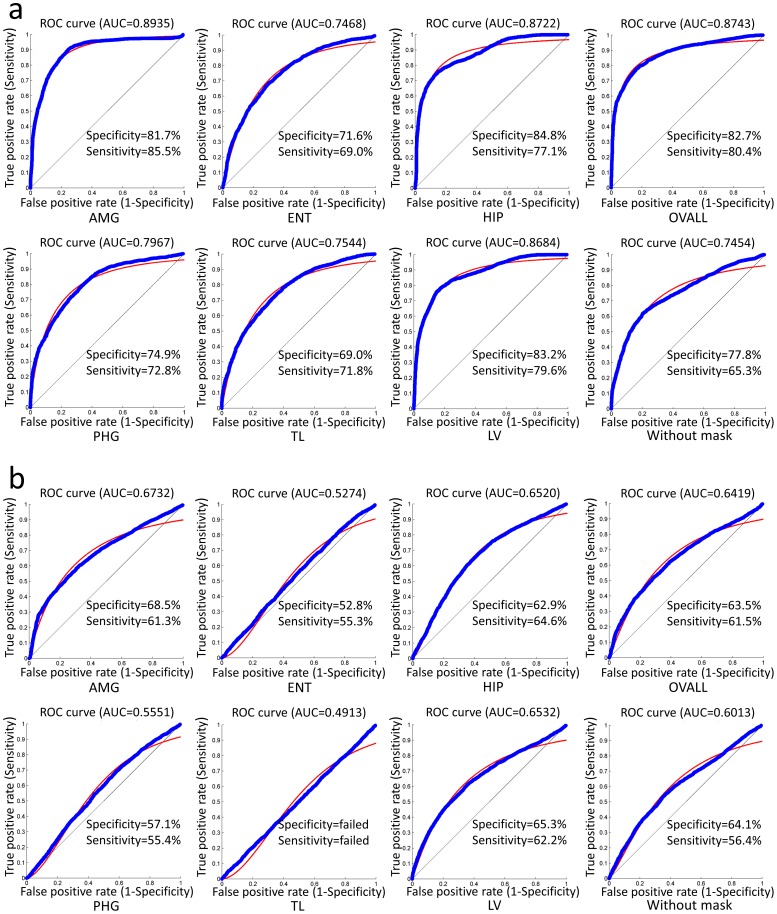
Classification performances with respect to features selected by seven masks in differentiating a) AD from NC, and b) MCI from NC. The performances were measured in terms of specificity, sensitivity, and AUC. The ROC curves are also displayed in the blue color with a smooth fitting line shown in red.

To bring anatomical knowledge into the present features, seven custom masks were used as priors for the second-round feature selection. The classification performance with respect to each mask was evaluated through 10-fold cross-validation, and the accuracies are displayed in Fig 5. Taking AUC as the standard measurement, the discriminative power in AD vs. NC is AMG>OVALL>HIP>LV>PHG>TL>ENT, whereas MCI vs. NC showed AMG>LV>HIP>OVALL>PHG>ENT>TL. The between-mask differences in the classification performance was evaluated using two-tailed McNemar's test [59]. As shown in Table 2, in AD vs. NC, no significant differences (p>0.05) were found in classification performance among classifiers using OVALL, AMG, and HIP. In MCI vs. NC, no significant differences (p>0.05) were found in classification performance among classifiers using OVALL, AMG, HIP, and LV. Note that the p-value was converted from the z-scores according to a z-score lookup table where z>1.960 corresponds to P<0.05. In addition, the performances of the outputs from first-step selection are also shown in Fig 5 under the caption “without mask.” In general, the features that underwent the two-step feature selection performed better than the features that underwent the first-step selection alone, in both AD vs. NC and MCI vs. NC.

**Table 2:** The between-mask differences of the classification performance evaluated by a two-tailed McNemar's test.

AD vs. NC							
	OVALL	AMG	ENT	HIP	PHG	TL	LV
**OVALL**	-Inf	0.42	19.56	0.03	14.17	21.35	3.56
**AMG**	0.42	-Inf	18.65	0.48	13.61	18.22	3.27
**ENT**	19.56	18.65	-Inf	21.68	9.85	1.54	17.19
**HIP**	0.03	0.48	21.68	-Inf	15.00	20.16	3.56
**PHG**	14.17	13.61	9.85	15.00	-Inf	5.77	10.73
**TL**	21.35	18.22	1.54	20.16	5.77	-Inf	18.06
**LV**	3.56	3.27	17.19	3.56	10.73	18.06	-Inf

MCI vs. NC							
	OVALL	AMG	ENT	HIP	PHG	TL	LV
**OVALL**	-Inf	3.77	14.10	1.89	11.38	19.92	2.49
**AMG**	3.77	-Inf	16.24	1.40	14.85	20.97	1.32
**ENT**	14.10	16.24	-Inf	15.95	3.12	4.10	15.06
**HIP**	1.89	1.40	15.95	-Inf	12.24	18.82	0.22
**PHG**	11.38	14.85	3.12	12.24	-Inf	7.03	13.37
**TL**	19.92	20.97	4.10	18.82	7.03	-Inf	19.65
**LV**	2.49	1.32	15.06	0.22	13.37	19.65	-Inf

The difference between every two masks was quantified in terms of z-scores. A threshold of z>1.960 was set to find the value beyond the 95% confidence level, which would be equal to p<0.05. In other words, the value z<1.960 indicates no significant difference. OVALL: combined mask. AMG: amygdala. ENT: entorhinal. HIP: hippocampus. PHG: parahippocampal gyrus. TL: temporal lobe. LV: lateral ventricle.

### Comparison of three types of features

Three types of features were trained and tested separately by SVM through 10-fold cross-validation. Their classification accuracies were shown in Table 3 in terms of sensitivity, specificity, AUC, and accuracy rate. Among all models, the one based on type 3 features performed best in all measurements. In addition, the results have proven that features directly extracted from unadjusted gray-scale images from multiple scanners were not usable since this yielded an ROC curve partly under the random guess line (a line connecting point [0 0] and point [1 1] in ROC space). The difference in the performance between type2 and type3 features was measured with a one-tailed McNemar's test. A significant difference was found in differentiating MCI from NC (p<0.05, z = 14.21), as well as in differentiating AD from NC (p<0.05, z = 3.87).

**Table 3:** The classification accuracies with respect three types of features, including type1) intensity-unadjusted image, type2) histogram-equalized image, and type3) LBP-TOP maps.

		accuracy	specificity	senSitivity	auc
**AD VS. NC**	TYPE1	63.09%	FAILED	FAILED	FAILED
	TYPE2	80.98%	81.5%	74.5%	0.843
	TYPE3	82.84%	82.7%	80.4%	0.874
**MCI VS. NC**	TYPE1	43.27%	FAILED	FAILED	FAILED
	TYPE2	52.93%	50.00%	55.20%	0.529
	TYPE3	61.53%	63.50%	61.50%	0.642

The performances are shown in in terms of sensitivity, specificity, AUC, and accuracy rate. Specificity, sensitivity and AUC are not shown for failed tests.

### Comparison of computation time

The proposed framework was compared with several widely used pipelines using different tools, such as SPM, FSL, HAMMER, AIR, LDDMM, and Freesurfer [44], [60]–[62] (Table 4). No considerable differences were shown in time consumption for model training or feature extraction among all methods, compared to the difference in the time spent on the preprocessing phase. For example, the duration of model training when managing 400 samples with each sample characterized by a feature vector containing 2.7e+5 variables (often the selected voxels) lasts for several minutes, compared with several seconds when managing the same number of samples with dozens or hundreds of variables in each feature vector (often the ROI-based features). However, time spent on non-rigid registration can be what potentially determines computation time of the whole pipeline, which varies from seconds to days.

**Table 4:** Computation time of the proposed model, together with computation times of several widely used pipelines using different tools like SPM, FSL, HAMMER, AIR, LDDMM, and Freesurfer.

Method type	Co-registration (per subject)	Segmentation Non-rigid registration (per subject)	Training	Testing (per subject)
**VBM**	SPM, <1 minute	SPM, >10 minutes	Minutes	>10 minutes
**Volume/shape**	FSL, Minutes	HAMMER, Hours	Seconds	Hours
	AIR, Seconds	LDDMM, Hours	Seconds	Hours
**Thickness**	Freesurfer, Days		Seconds	Days
**Proposed method**	SPM, <1 minute	/	Minutes	< 1minutes

## Discussion

Here, we propose an efficient approach to differentiate AD or MCI from NC, based on multicenter MRI using gray-level invariant features. Before the discussion of discriminative powers, biological sensitivities of features were studied as part of the demonstration of their feasibilities. It can be seen from the results that top-ranked areas that have greatest discriminative power include the hippocampus, the amygdala, the anterior/posterior cingulate gyrus, and several areas in temporal lobe. These areas agree well with previous findings of gray-matter loss in temporal-limbic regions, as well as in anatomically associated regions like the cingulate gyrus, and the precuneus [63]–[65]. Significant differences were also seen in other areas that were within or around the lateral ventricles, possibly related to ventricle enlargement and its joint influence in some deep gray matter areas like the thalamus and putamen [66], [67]. Since the current study is based on rigid transformation only, the structural changes shown in the images encode not only non-rigid information, but also some rigid information. For example, atrophy of a certain area might pull/shift its neighboring tissues. Therefore, the selected features might not be right inside those accepted areas associated with AD.

To further validate the effectiveness of proposed features, especially the robustness to cross-scanner variability, the discriminative value was studied by measuring the performance in differentiating AD/MCI from NC subjects retrieved from multiple institutes, and then compared with accuracies with respect to features based on intensity-unadjusted images and histogram-equalized images based on the same database. Without doubt, features based on intensity-unadjusted images were practically useless due to the non-robustness to cross-scanner variability of training samples. The histogram-equalized image encodes feasible features for the classification of AD and NC (AUC>0.8). This method assigned 80.98% of the subjects to the correct category, although resulting in lower accuracy in terms of all measurements than proposed features. However, performance with respect to histogram equalization is less effective in differentiating MCI and NC (AUC<0.6), compared to the proposed features that produced AUC>0.6.

Compared with results reported in previous literature using voxel, volume, and thickness features in AD vs. NC discrimination, the proposed features (using the top-ranked mask AMG) produced a sensitivity higher than 81.0% [68], 85.0% [69], and 85.0% [70], but lower than 86.0% [71]; and a specificity higher than 80.0% [69], but lower than 95.0% [68], 86.3% [71], and 93.0% [70]. In MCI vs. NC, the proposed method (using the top-ranked mask AMG) yielded a sensitivity lower than 73.0% [68], 78.5% [71], 84.0% [70]; and a specificity higher than 59.6% [71], but lower than 85.0% [68], and 86.0 [70]. However, the direct comparison of performance between the proposed method and previous methods could be only for reference because these approaches were based on different subgroups of ADNI datasets or variant strategies of cross-validation. In addition, models in these previous studies were also trained and tested using different classifiers; for instance, LP boosting was employed in [69] and LDA was employed in [70]. Parts of these studies also used a hierarchical fusion classifier with features from multimodal imaging techniques [71]. Overall, LBP -based features performed well in AD vs. NC classification, but was less effective in MCI vs. NC tasks compared to the approaches based on sophisticated measurements of multiple brain tissues. A possible explanation is that MCI patients showed very subtle structural changes that could be captured only by high-dimensional spatial normalization. We would like to stress that our approach does not necessarily compete with or replace more traditional approached based on non-linear transformation. Although it is obvious that non-linear transformation is needed to achieve better image registration, non-linear registration also invites nuisance factors, including dependence on transformation algorithms, cost functions, and employed parameters. If there is a large anatomical difference between the atlas and patient images, there is always a chance to be trapped in a local minima. Because the cost functions are usually based on image intensities, non-linear approaches are sensitive to contrast differences, and thus, potentially to protocol differences. Having only a limited number of solutions, the linear solutions are more robust against these factors. It is, therefore, a reasonable approach to test features extraction based on the linear solution before resorting to the non-linear solution. This result could form a foundation on which to judge the efficacy of non-linear solutions; for example, the linear solution can be used as a benchmark to evaluate the improved sensitivity and specificity to identify a patient group. Alternatively, the time-efficient linear solution could be used as pre-processing for initial stratification and anatomical homogenization, such that the non-linear solutions would be more reliable. The linear solution, on the other hand, invites its own complications, especially in data interpretation. We can no longer assume that each defined structure in the atlas accurately identifies the target structure in the subject, and, thus, the measured intensity is not the intensity of the target structure per se, but reflects the amount of mis-registration due to anatomical variability. This unique features extraction approach requires us to design and test an appropriate statistical approach.

The present method can be used as a tool for fast recognition of anatomic features with guidance based on the criteria from the neurological diagnosis. A natural extension of the proposed method is an automated image categorization tool to assist clinical decision-making, since it yielded reliable sensitivity and specificity when differentiating AD from NC. The performance of the automated categorization could be further improved when combined with other laboratory data, such as the examination of cerebrospinal fluid (CSF), or other imaging modalities, such as PET. Studies based on pathologically diagnosed cases are expected to play important roles in establishing the usefulness of automated image categorization in diagnosis and clinical decision-making.

The purpose of our current study was to offer an alternative to a voxel-based approach to capture valuable anatomical information results, with respect to AD or MCI based on a linear transformation. Although the proposed technique was validated in this study to demonstrate feasible classification, it is still lacking in framework for the individual risk assessment for eventually developing AD. In line with this, our future work involves the development of an AD prediction model from mild cognitive impairment converters (MCI-c). In addition, the present work is based on features extracted on LBP-TOP maps only. Thus, comparative studies of the effectiveness of models that employ other forms of 3D LBP [72], [73] are highly anticipated in the future.

In conclusion, the present approach directly encodes disease-specific patterns on a voxel-wise basis without non-rigid registration. Owing to its computational efficiency, along with its characteristic of gray-level invariance, the proposed approach could be a useful tool for the analysis of large medical image data and also could be a supplementary method to more detailed subsequent analyses based on non-linear transformation
